# One Sensible Man

**Published:** 1871-02

**Authors:** 


### ONE SENSIBLE MAN

Lives away down in beautiful Tennessee, who, whenever he has a new guest, takes an early occasion to show him around his premises; the parlor, the library, the bath-room, his own apartment while a guest, and last, not least, the privy or water-closet, which is among the most faultlessly tidy portions of his establishment. His neighbors say of him sometimes he is "queer in some things." Pity is it that there are not a million to one like him. About ten years ago this very point was brought to the attention of the readers of the Journal, not one of whom could fail to see, nor can now fail to see the delicacy, wisdom, and healthfulness of the idea. How furtively has the reader, many a time, on going to a strange place looked around for a servant, and with averted eyes and whispering tone made inquiry as to this point; and then again how much afraid of encountering the opposite sex in going or returning, to say nothing of a most disagreeable apprehension of intrusion or interruption.

On farms and on all country places, it should not fail to be arranged to have what is necessary in connection with the stable; for among other reasons a gentleman is naturally supposed to have an interest in horses, and they could saunter around in that direction, those who saw them supposing they were going to look at the noblest of animals, while the man himself persuades his own mind that any one seeing him would suppose he was visiting the stable to take a look at its arrangements and occupants, so subtle a being is man.

Constipation and piles are brought on in innumerable cases by deferring the calls of nature for the above or other causes, and standard medical works give detailed cases where persons by delays have induced inflammation of the bladder, dying in three or four days. Every mother should consider it a duty to impress upon the minds of children, beginning at the age of three or four years, the danger of delaying the calls of nature, and the influence it has in imparing the health, and in bringing on life-long ailments.

Almost all our common ailments are attended with costiveness, and as the bowels become free again, recovery takes place. If a mother were to draw the attention of a sick child to this fact that when the bowels began to act, the health improved, and that when sick the bowels had failed to act that day and perhaps the day before, it would make a practical and a radical impression on the child's mind, with life-long benefits.

